# A multi-omics study links *TNS3* and *SEPT7* to long-term former smoking NSCLC survival

**DOI:** 10.1038/s41698-021-00182-3

**Published:** 2021-05-17

**Authors:** Sipeng Shen, Yongyue Wei, Yi Li, Weiwei Duan, Xuesi Dong, Lijuan Lin, Dongfang You, Adonina Tardon, Chu Chen, John K. Field, Rayjean J. Hung, Geoffrey Liu, Dakai Zhu, Christopher I. Amos, Li Su, Yang Zhao, Zhibin Hu, Hongbing Shen, Ruyang Zhang, Feng Chen, David C. Christiani

**Affiliations:** 1 Department of Biostatistics, Center for Global Health, School of Public Health, Nanjing Medical University, Nanjing, 211166 Jiangsu China; 2 State Key Laboratory of Reproductive Medicine, Nanjing Medical University, Nanjing, 211166 Jiangsu China; 3 China International Cooperation Center of Environment and Human Health, Nanjing Medical University, Nanjing, 211166 Jiangsu China; 4 Department of Biostatistics, University of Michigan, Ann Arbor, MI 48109 USA; 5 Department of Bioinformatics, School of Biomedical Engineering and Informatics, Nanjing Medical University, Nanjing, 211166 Jiangsu China; 6 Department of Environmental Health, Harvard T.H. Chan School of Public Health, Harvard University, Boston, MA 02115 USA; 7 University of Oviedo and CIBERESP, Faculty of Medicine, Oviedo, 33003 Spain; 8 Program in Epidemiology, Division of Public Health Sciences, Fred Hutchinson Cancer Research Center, Seattle, WA 98109 USA; 9 Institute of Translational Medicine, University of Liverpool, Liverpool, UK; 10 Prosserman Centre for Population Health Research, Lunenfeld-Tanenbaum Research Institute, Sinai Health System and University of Toronto, Toronto, ON M5T 3L9 Canada; 11 Princess Margaret Cancer Centre, Toronto, ON M5G 2C1 Canada; 12 Department of Medicine, Epidemiology Section, Institute for Clinical and Translational Research, Baylor Medical College, Houston, TX 77030 USA; 13 Department of Epidemiology, Center for Global Health, School of Public Health, Nanjing Medical University, Nanjing, 211166 Jiangsu China; 14 Jiangsu Key Lab of Cancer Biomarkers, Prevention and Treatment, Cancer Center, Collaborative Innovation Center for Cancer Personalized Medicine, Nanjing Medical University, Nanjing, 211166 Jiangsu China; 15 Pulmonary and Critical Care Division, Massachusetts General Hospital, Department of Medicine, Harvard Medical School, Boston, MA 02114 USA

**Keywords:** Prognostic markers, Cancer epigenetics

## Abstract

The genetic architecture of non-small cell lung cancer (NSCLC) is relevant to smoking status. However, the genetic contribution of long-term smoking cessation to the prognosis of NSCLC patients remains largely unknown. We conducted a genome-wide association study primarily on the prognosis of 1299 NSCLC patients of long-term former smokers from independent discovery (*n* = 566) and validation (*n* = 733) sets, and used in-silico function prediction and multi-omics analysis to identify single nucleotide polymorphisms (SNPs) on prognostics with NSCLC. We further detected SNPs with at least moderate association strength on survival within each group of never, short-term former, long-term former, and current smokers, and compared their genetic similarity at the SNP, gene, expression quantitative trait loci (eQTL), enhancer, and pathway levels. We identified two SNPs, rs34211819_*TNS3*_ at 7p12.3 (*P* = 3.90 × 10^−9^) and rs1143149_*SEPT7*_ at 7p14.2 (*P* = 9.75 × 10^−9^), were significantly associated with survival of NSCLC patients who were long-term former smokers. Both SNPs had significant interaction effects with years of smoking cessation (rs34211819_*TNS3*_: *P*_*interaction*_ = 8.0 × 10^−4^; rs1143149_*SEPT7*_: *P*_*interaction*_ = 0.003). In addition, in silico function prediction and multi-omics analysis provided evidence that these QTLs were associated with survival. Moreover, comparison analysis found higher genetic similarity between long-term former smokers and never-smokers, compared to short-term former smokers or current smokers. Pathway enrichment analysis indicated a unique pattern among long-term former smokers that was related to immune pathways. This study provides important insights into the genetic architecture associated with long-term former smoking NSCLC.

## Introduction

Non-small cell lung cancer (NSCLC) accounts for more than 85% of lung cancer cases and is the most commonly diagnosed malignant disease^1^. Tobacco smoking is a well-known environmental exposure leading to lung cancer^2,3^ and has been found to be linked to the majority of NSCLC deaths^4^. Emerging evidence shows that NSCLC in smokers and never smokers are different and separate entities^5–7^.

It is, however, less well known that former smokers who quitted smoking long time before (e.g., >10 years), termed long-term former smokers, may also develop lung cancer^8^, and NSCLC patients who are long-term former smokers still harbor a high mortality risk^9^. So far, few studies have focused on the impact of genetic architecture on the prognosis of such a special group of patients, whose molecular mechanism for cancer death still remains unclear.

Leveraging the well-established International Lung Cancer Consortium (ILCCO) and Harvard Lung Cancer Study (HLCS), we conducted a GWAS by focusing on NSCLC patients who were long-term former smokers and identified germline genetic variants associated with overall survival time. We detected a set of SNPs with at least moderate association signals with survival from this subgroup as well as from other smoking subgroups, namely, never smokers, short-term former smokers, and current smokers. Comparing these SNPs, we investigated the genetic similarity across these smoking subgroups at the SNP, gene, expression quantitative trait loci (eQTL), enhancer, and pathway levels.

## Results

### Genetic variants are associated with survival

**Fig. 1:**
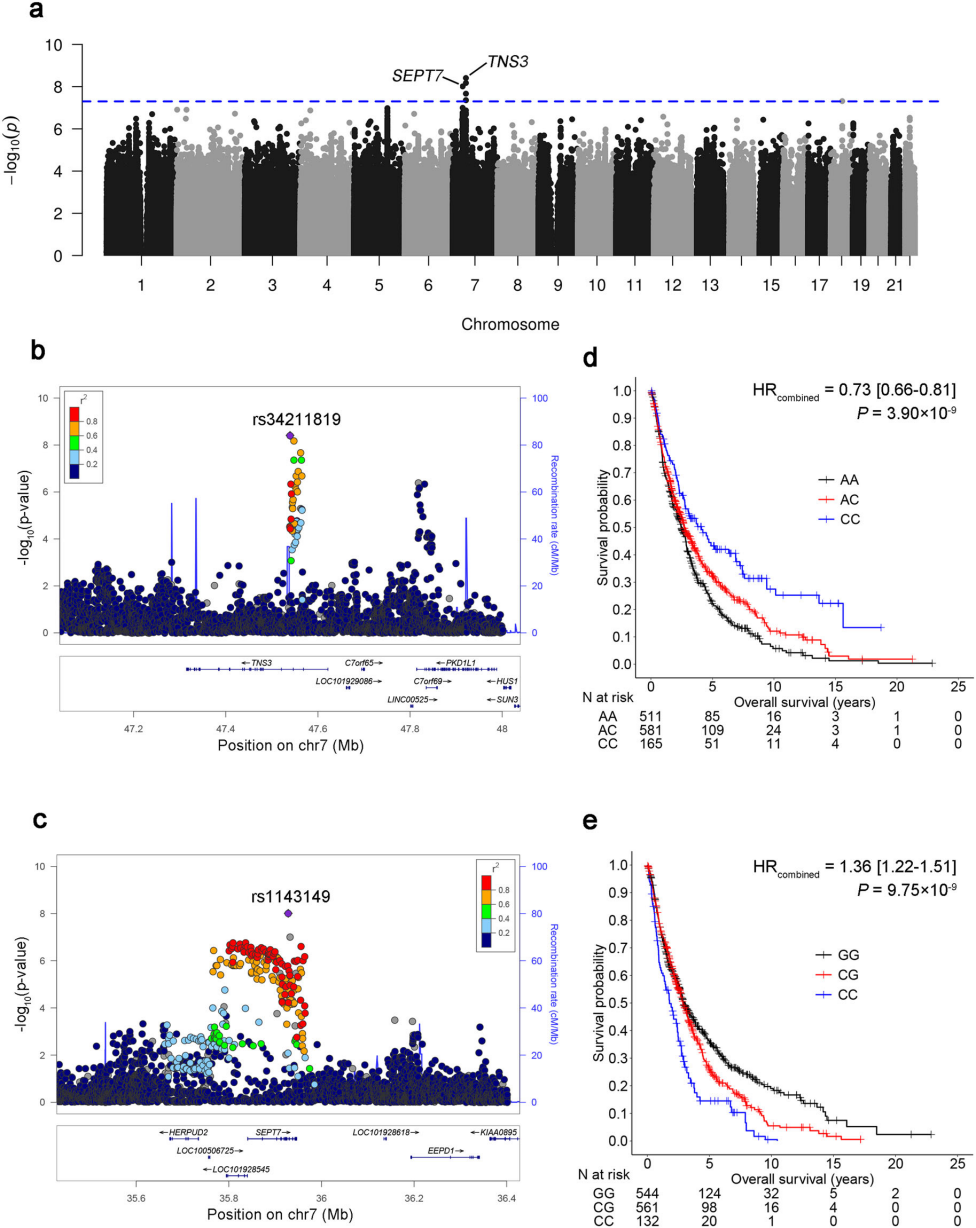
Results of GWAS survival analysis. **a** Manhattan plot for survival analysis *P* values. All genetic variants were coded using an additive model. SNPs with a minor allele frequency >5%, imputation *R*^*2*^ > 0.8, and Hardy–Weinberg equilibrium *P* > 1×10^−5^ were included. **b**, **c** Regional association plots for rs34211819 in *TNS3* and rs1143149 in *SEPT7*. The left-hand Y-axis shows −log10 transformation of the *P*-value of individual SNPs plotted against the chromosomal base-pair position with an expansion of 500 kb in the flanks of the SNP position. The right-hand *Y*-axis shows recombination rate estimated for European populations from HapMap Data Rel 22/phase II. **d**, **e** Kaplan–Meier survival curves for rs34211819 and rs1143149 in NSCLC patients by 0, 1, and 2 minor alleles. HRs, 95% CI and *P* values were derived from Cox proportional hazards regression models adjusted for covariates.

**Fig. 2:**
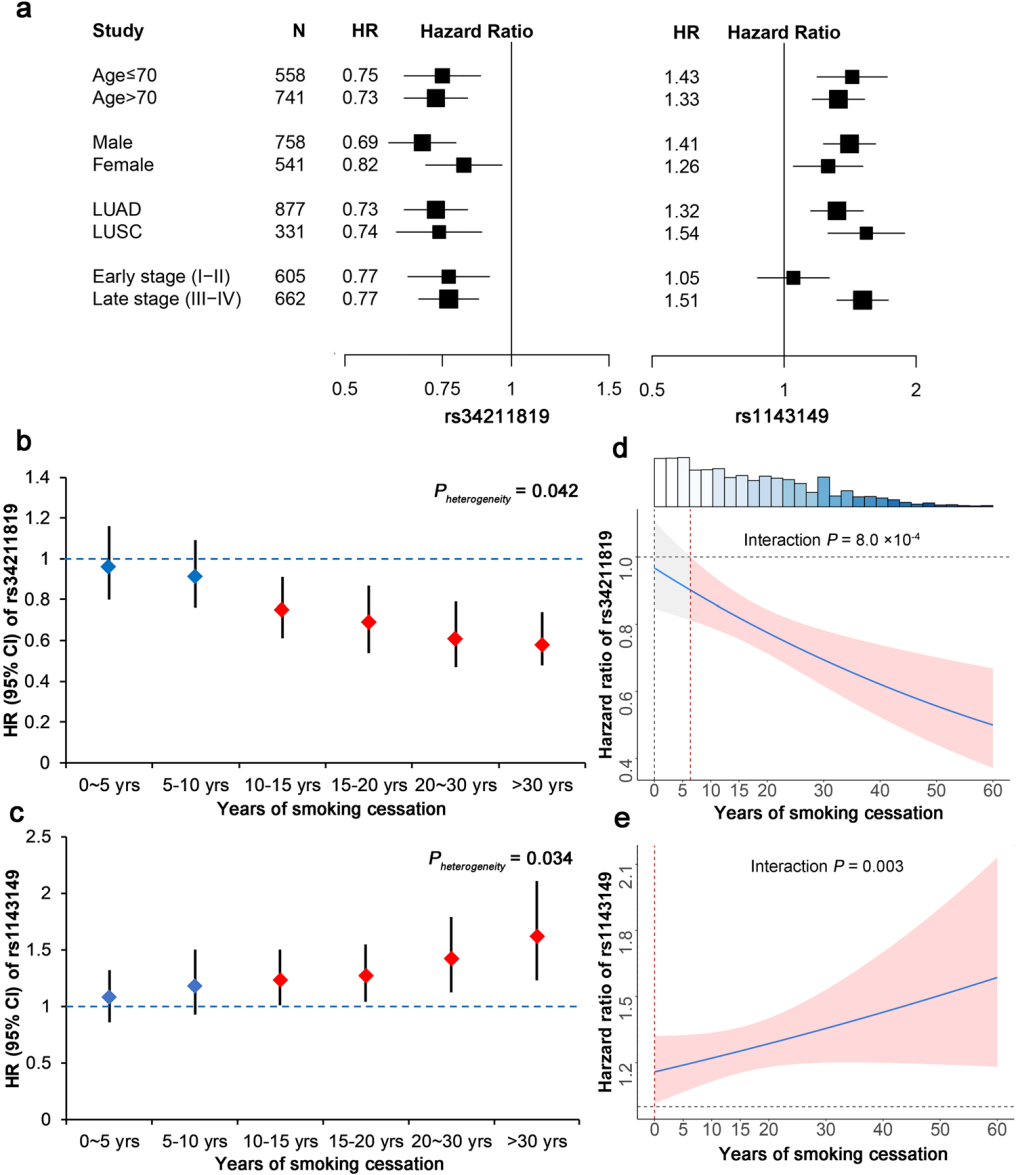
Stratified analysis and interaction analysis with smoking cessation years. **a** Stratified analysis of rs34211819 (left) and rs1143149 (right) among different subgroups of long-term former smokers by age (divided by median value), gender, histology type, and clinical stage. **b, c** Stratified analysis of rs34211819 and rs1143149 by different years of smoking cessation in overall NSCLC patients. The left-hand *Y*-axis shows the effect size (HR and 95% CI) derived from the Cox proportional hazards regression model. **d, e** Interaction plot for HRs of SNPs estimated based years of smoking cessation. The shaded area represents the 95% CI. Top histogram shows distribution of years of smoking cessation.

The genome control inflation factor (*λ*) for the overall population under the additive model was estimated to be 1.079 (Supplementary Fig. 1), which was comparable to a previous genome-wide survival study^10^, indicating that the confounding effect caused by population stratification was well controlled. Two SNPs (rs34211819 at chromosome 7p12.3, and rs1143149 at 7p14.2) reached genome-wide significance among NSCLC patients who were long-term former smokers (Fig. 1a). rs34211819 was in an intron region of tensin-3 (*TNS3*), with an MAF of 0.35; rs1143149 was an intron variant of septin 7 (*SEPT7*), with an MAF of 0.34. The regional plots (Fig. 1b, c) present a cluster of significant prognostic SNPs that were moderately or highly correlated with rs34211819 and rs1143149 (Supplementary Table 1). Although the chromosome locations of the two SNPs were close, they had a very low correlation (*D*’ = 0.017, *r*^*2*^ = 2.27 × 10^−5^). The C allele of rs34211819 was significantly associated with better survival (HR_*discovery*_ = 0.66, 95% CI: 0.56-0.78, *P* = 2.01 × 10^−7^; HR_*validation*_ = 0.84, 95% CI: 0.73–0.96, *P* = 1.36 × 10^−2^; *HR*_*combined*_ = 0.73, 95% CI: 0.66–0.81, *P* = 3.90 × 10^−9^) (Fig. 1d). In contrast, the C allele of rs1143149 was associated with worse survival (HR_*discovery*_ = 1.42, 95% CI: 1.16–1.60, *P* = 2.04 × 10^−6^; HR_*validation*_ = 1.33, 95% CI: 1.15–1.53, *P* = 7.88 × 10^−5^; *HR*_*combined*_ = 1.36, 95% CI: 1.22–1.51, *P* = 9.75 × 10^−9^) (Fig. 1e). The associations remained significant if SNPs were coded in co-dominant, dominant, and recessive models (Supplementary Table 2), indicating the robustness of our results toward coding. The same conclusion held with analyses of subgroups, defined by age, gender, histology, and clinical stage (Fig. 2a), as both SNPs were significantly associated with survival in almost all subgroups, except for rs1143149 among the early-stage patients.

### Genetic variants interaction with years of smoking cessation

We performed a stratified analysis by years of smoking cessation for both long-term and short-term former smokers to evaluate the modifying effect of years of smoking cessation. As years of smoking cessation increased, the protective effect of rs34211819 and the detrimental effect of rs1143149 on survival were both elevated (Fig. 2b, c). These effects of two SNPs were only significant in long-term former smokers, and not in short-term former smokers, indicating significant heterogeneity between these two groups (rs34211819: *P*_*heterogeneity*_ = 0.042; rs1143149: *P*_*heterogeneity*_ = 0.034). The trend test detected significant trends for both SNPs across different subgroups (rs34211819: *P*_*trend*_ = 0.003; rs1143149: *P*_*trend*_ = 0.045). Further, we detected significant interaction effects between SNPs and years of smoking cessation, a type of gene–environment interactions (rs34211819 × years: *P*_*interaction*_ = 8.0 × 10^−4^; rs1143149 × years: *P*_*interaction*_ = 0.003) (Fig. 2d, e).

### Evidence of association with lung cancer survival from multi-omics studies

**Fig. 3:**
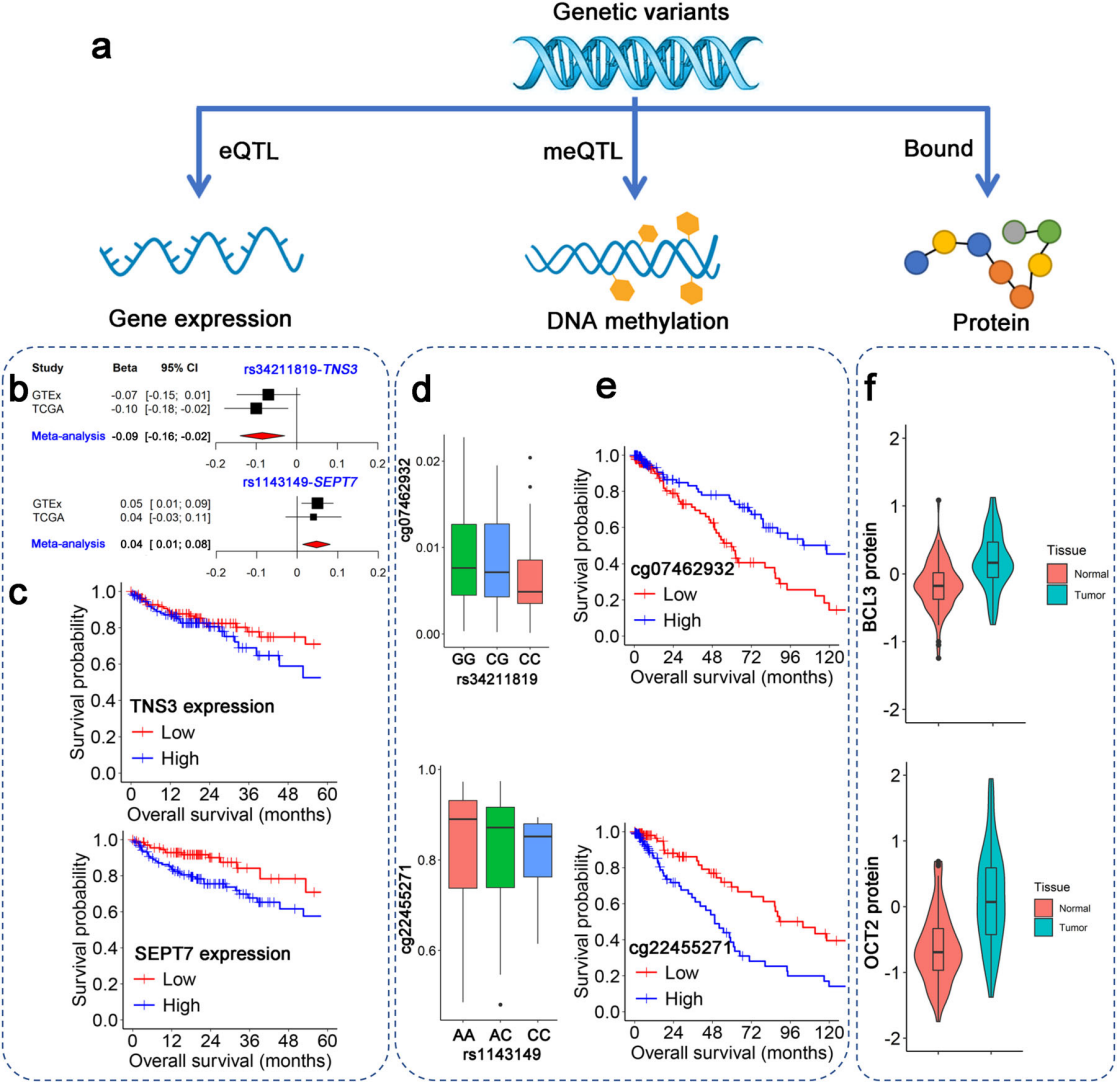
Multi-omics analyses for *TNS3* and *SEPT7*. **a** Multi-omics analyses flowchart. We observed significant eQTL/meQTL relationship, survival-related methylation, and expression patterns and upregulated proteins in NSCLC. **b** Significant eQTLs were observed for the two SNPs and cis genes. **c** Kaplan–Meier survival curves for expression of both genes using TCGA long-term former smoking patients. **d** meQTL boxplots of the two CpG probes and SNPs. **e** DNA methylation survival analysis for the two CpG probes. **f** Proteins BCL3 and OCT2 bound by SNPs also differed between tumor and normal tissues.

In meta-analysis of eQTL effects from GTEx and TCGA, the two SNPs had a significant cis-eQTL relationship with gene expression (*TNS3*: *β* = −0.09, 95% CI: −0.16 to −0.02, *P* = 0.009; *SEPT7*: *β* = 0.04, 95% CI: 0.01 to 0.08, *P* = 0.033) (Fig. 3b). In gene expression survival analysis of long-term former smokers, higher expression of *TNS3* (*HR* = 1.84, 95% CI: 1.01–3.34, *P* = 0.045) and *SEPT7* (*HR* = 1.67, 95% CI: 1.02–3.21, *P* = 0.023) were significantly associated with worse survival in the TCGA database (Fig. 3c).

In DNA methylation analysis, we extracted 94 CpG probes that were located within *TNS3* and *SEPT7*. Two CpG sites, cg22455271 and cg07462932, had significant methylation QTL (meQTL) effects with rs1143149 (*β* = -0.08, 95% CI: −0.14 to −0.01, *P* = 0.009, *q*-FDR = 0.034) and rs34211819 (*β* = −0.17, 95% CI: −0.32 to −0.02, *P* = 0.002, *q*-FDR = 0.039), respectively (Fig. 3d). They were significantly associated with survival in long-term former smokers (HR_cg22455271_ = 2.40, 95% CI: 1.47–3.93, *P* = 2.5 × 10^−4^, *q*-FDR = 0.001; HR_cg07462932_ = 0.47, 95% CI: 0.30–0.74, *P* = 7.4 × 10^−4^, *q*-FDR = 0.018) (Fig. 3e and Supplementary Table 3).

We predicted the functional relevance by SNPinfo, RegulomeDB, and HaploReg v4.1 (Supplementary Table 4). rs34211819 in *TNS3* had a high score of protein binding and could bind two proteins: B cell lymphoma 3 (BCL3) and octamer transcription factor 2 (OCT2). In the CPTAC proteomics project, the two bound proteins BCL3 (fold change = 1.69, *P* = 2.16 × 10^−5^) and OCT2 (fold change = 2.21, *P* = 3.13 × 10^−13^) were significantly upregulated in the lung cancer tumor tissues compared to the adjacent normal tissues (Fig. 3f).

### Genetic similarity across patients with different smoking statuses

**Fig. 4:**
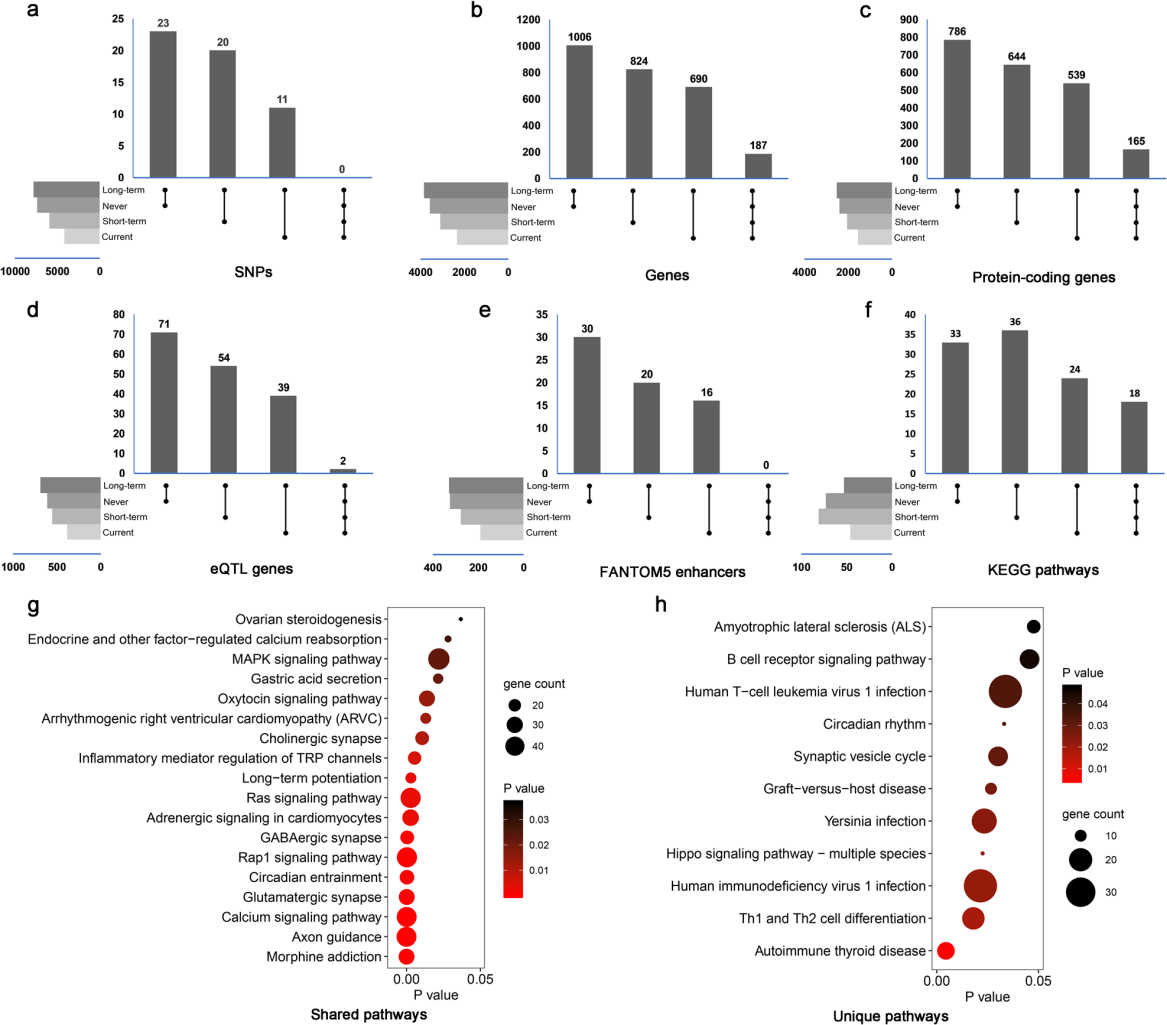
Results of genetic similarity comparative analysis. Similarity comparison of SNPs with *P* < 10^−3^ at SNP level (**a**), germline-regulated gene level (**b**), germline-regulated protein-coding gene level (**c**), eQTL-related gene level (**d**), enhancer level (**e**), and KEGG pathway level (**f**), respectively. Protein-coding genes were defined by the GENCODE database. **g–h** Shared and unique KEGG pathways in long-term former smokers compared with never, short-term former, and current smokers.

We first performed genetic similarity comparisons at the SNP level by extracting moderate-to-high signals from the results of survival analysis within different smoking subgroups. A total of 7789 independent SNPs was observed in the long-term former smokers, which were comparable to that of never-smokers (*n* = 7358) but 31.4% and 85.5% more than that of short-term former smokers (*n* = 5343) and current smokers (*n* = 4198), respectively. No SNPs were shared across the four smoking subgroups. Long-term former smokers only had 23, 20, and 11 overlapping SNPs with never smokers, short-term former smokers, and current smokers, respectively (Fig. 4a), indicating that these significant prognostic SNPs for NSCLC patients who were long-term former smokers seemed to differ from those for the other smoking groups.

For gene-level comparisons, these identified SNPs were assembled to genes within each subgroup (Fig. 4b). We identified 3,837 genes in long-term former smokers, and 1006 genes of them were shared with never-smokers, 22.1% more than those shared with short-term former smokers (824 genes) and 45.8% more than with current smokers (690 genes). A total of 187 genes were commonly shared by all the subgroups (Supplementary Table 5). Protein-coding genes showed these same patterns of similarity (Fig. 4c).

We further investigated the eQTL-related genes based on the GTEx lung tissue database (Fig. 4d) and enhancers from the FANTOM5 database (Fig. 4e). For long-term former smokers, the same trend was observed with germline-regulated genes, which may indicate the higher similarity with never-smoking subgroup than others. Only two eQTL-related genes were shared among all subgroups: *ARHGAP15* and *TSPAN9*. We also performed sensitivity analysis under different thresholds (*P* < 10^−4^ and *P* < 10^−5^) and obtained the similar results (Supplementary Table 6).

### Unique and shared pathways in long-term former smokers

We explored Kyoto Encyclopedia of Genes and Genomes (KEGG) pathways in the gene set enrichment analysis of germline-regulated genes. A total of 53 pathways were significant in long-term former smokers (Fig. 4f). A total of 18 pathways were shared across all the subgroups, including the well-known signaling pathways such as mitogen-activated protein kinase, oxytocin, Ras, Rap1, and calcium pathways (Fig. 4g). However, 11 pathways were only significant in long-term former smokers, most of which were linked to immune function such as the B cell receptor signaling pathway, human T cell leukemia virus 1 infection, human immunodeficiency virus 1 infection, and T helper type 1 and T helper type 2 cell differentiation (Fig. 4h).

## Discussion

Understanding genetic risk factors in cancer is important for uncovering its underlying biological mechanisms^11,12^. Our genome-wide investigation among long-term former smokers with NSCLC detected rs34211819 in *TNS3* and rs1143149 in *SEPT7* which were associated with survival. Significant interactions revealed that the effects of SNPs could be modified by years of smoking cessation, perhaps indicating opportunities for clinical adjuvant therapy with immunotherapies in patients with risk alleles, while an immune function relationship was found for *cis*-regulated genes or SNP-binding proteins. Multi-omics analyses provided evidence of their eQTL/meQTL relationships, survival-related methylation, and expression patterns and upregulated proteins in NSCLC. Furthermore, we observed higher similarities between long-term former smokers and never-smokers, compared to short-term former smokers or current smokers. The distinct SNP patterns in long-term former smokers were linked to immune signals.

Tobacco smoking is associated with worse outcomes in lung cancer, as it leads to downregulation of proinflammatory cytokines, immunosuppression, and anti-inflammatory effects mediated by oxidants, carbon monoxide, nicotine, and transcriptional modifying compounds, especially in lung tissues^13,14^. Chronic inhalation of cigarette smoke affects a wide range of immunological functions, including innate and adaptive immune responses^15^. As a result, the immune system may be suppressed as long as individuals are exposed to smoking regardless of years of quitting or smoking, as evidenced by the same signaling pathways shared by all the smoking subgroups in our study. Tobacco smoking can affect the immune system by chemically modifying signaling pathways as well as the extracellular matrix through acetylation, nitrosylation, carbonylation, and oxidation, thereby affecting cell survival, activation, and differentiation^16^.

The identified genes were associated with NSCLC survival at the genomic, epigenomic, and transcriptomic levels and were related to immunological functions. *SEPT7* is a member of the septin family of GTP-binding proteins, which form higher-order filamentous structures and function primarily in spatial organization and compartmentalization of many cellular processes*. SEPT7* is structurally related to *RAS* oncogenes, which promote tumorigenesis^17^. *SEPT7* is also implicated in several types of cancer^18–20^. While the septin family plays a critical role in cytokinesis^21^, *SEPT7* is also involved in the cytoskeleton and participates in regulation of cytokinesis^22,23^. Septin-deficient T cells fail to complete cytokinesis when prompted by pharmacological activation or cytokines. Meanwhile, *SEPT7*-deficient fibroblasts display incomplete cytokinesis and constitutive multinucleation by affecting the mitotic spindle and midbody rather than the contractile ring^24^. As a result, *SEPT7* plays a crucial role in immune functions including cytokinesis and mitosis, which are closely related to molecular changes from smoking exposure.

Another locus at 7p12 is marked by an intronic SNP in *TNS3*, which encodes tensin-3, a member of a family of focal adhesion-associated proteins that regulate cell adhesion and migration^25^. This gene may be an activator of cell migration and a promoter of invasion in tumor metastasis^26,27^. Here, we found that *TNS3* acts as an oncogene, affecting regulation of methylation in lung cancer. A *TNS3* methylation pattern in the promoter region can silence expression in renal cell carcinoma^28^. Further, the binding proteins BCL3 on 19q13.32 and OCT2 on 19q13.2 of rs34211819 are strongly linked to immune function. *BCL3* translocates to the immunoglobulin alpha-locus in B cell chronic lymphocytic leukemia^29^. As an oncogene, it is an atypical member of the inhibitor of nuclear factor kappa B (NF-κB) family of proteins that can activate the NF-κB signaling cascade by directly binding to the transcription factors NFKB1 and NFKB2^30^. It is unregulated by cytokines such as tumor necrosis factor alpha, interleukin 4 (IL-4), IL-1, and IL-6^31^. OCT2 acts as a DNA-binding transcriptional activator of immunoglobulin in B-lineage cells^32^. It enables B cells to respond normally to antigen receptor signals and mediate the physical interaction with T cells or to produce and respond to cytokines that are critical drivers of B cell and T cell differentiation during the immune response^31,33^.

Genetic similarity analysis of subgroups showed lower overlap at the SNP level but relatively higher overlap of germline-regulated genes. It is possible that each SNP is unique to each subgroup and acts as an eQTL to regulate cancer-specific genes. Additionally, the effects of somatic mutations driving lung cancer should be investigated. We also found higher similarities between long-term former smokers and never-smokers, which mainly included inflammation and immune mechanisms, as reported in the previous studies^10,34^.

This study had some limitations. First, although we used the largest lung cancer consortium to date, further external cohort validation with follow-up information and smoking cessation details is warranted. Second, we selected SNPs with moderate association strengths (from *P* < 10^−5^ to *P* < 10^−3^); although it was a reasonable approach^35,36^, some false-positive SNPs may have been included. More well-designed functional experiments are necessary to validate the biological functions.

This study also had several strengths. To the best of our knowledge, this is the first GWAS to investigate the effects of genetic variants on NSCLC patients among long-term former smokers. We included a large and relatively homogeneous study population with relatively complete and accurate follow-up, demographic, and clinical covariate information from ILCCO and HLCS. In addition, we investigated the association of candidate genes and lung cancer at the multi-omics levels, including genomics, transcriptomics, epigenomics, and proteomics. Similarity comparisons among different smoking subgroups revealed the shared genetics status at multiple levels.

In summary, our study demonstrated that *TNS3* at 7p12.3 and *SEPT7* at 7p14.2 are genetic regions associated with survival among long-term former smokers, and the findings with subgroup were related to immune function. Our results may shed light on the important roles of genetic architecture on cancer outcomes among long-term former smokers with NSCLC, a subpopulation that has been less studied.

## Methods

### Study population

In accordance with the previous studies^37–39^, long-term former smokers were defined as patients who quitted smoking at least 10 years before diagnosis, whereas short-term former smokers quitted <10 years before diagnosis, and never smokers were those who smoked <100 cigarettes during their lifetime. To identify prognostic SNPs, we focused on long-term former smokers, whose characteristics are presented in Table 1. Patients in the discovery set were recruited from ILCCO, including the Cancer de Pulmon en Asturias study, Carotene and Retinol Efficacy Trial (CARET), Liverpool Lung Cancer Project, MD Anderson Cancer Center Study, and Mount-Sinai Hospital-Princess Margaret Study. While, patients in the independent validation set were recruited from HLCS (Supplementary Information). Approval for ILCCO studies was obtained from each of the participating institutional research ethics review boards. For HLCS, the Institutional Review Board of MGH and the Human Subjects Committee of the Massachusetts General Hospital and Harvard School of Public Health approved the study. All the participants were provided written informed consent to take part in the study.

**Table 1:** Demographic and clinical characteristics of long-term former smoking NSCLC patients.

Characteristics	Discovery set	Validation set	Combined set
Sample size	566	733	1,299
Deaths (%)	387 (68.4)	500 (68.2)	887 (68.2)
Median survival years (95% CI)	2.14 (1.71–2.51)	2.87 (2.69–3.31)	2.64 (2.38–2.86)
Age (years)	70.98 ± 8.79	70.01 ± 9.22	70.43 ± 9.04
Gender, male (%)	373 (65.9)	385 (52.5)	758 (58.4)
Histology (%)			
LUSC	194 (61.4)	137 (18.7)	331 (25.5)
LUAD	348 (34.2)	529 (72.2)	877 (67.5)
NSCLC, not specified	24 (4.2)	67 (9.1)	91 (7)
Clinical stage (%)			
I	207 (36.5)	258 (35.2)	465 (35.8)
II	68 (12.0)	72 (9.8)	140 (10.8)
III	131 (23.1)	165 (22.5)	296 (22.8)
IV	138 (24.3)	228 (31.1)	366 (28.2)
Pack-years of smoking	36.49 ± 29.00	37.49 ± 29.03	37.05 ± 29.01
Years of smoking cessation	23.18 ± 10.49	23.66 ± 10.39	23.45 ± 10.44

*LUAD* lung adenocarcinoma, *LUSC* lung squamous cell carcinoma, *NSCLC* non-small cell lung cancer.

Of the 6129 NSCLC patients with follow-up information totally, 4351 eligible cases were with available smoking information, including 504 never smokers, 1299 long-term former smokers, 687 short-term former smokers, and 1861 current smokers (Supplementary Table 7).

### OncoArray genotype quality control and imputation

The ILCCO study and HLCS were originally designed and genotyped as case–control studies of lung cancer risk. In this study, we extracted all NSCLC patients from these two studies with survival information. Patient genotypes were generated using the Infinium OncoArray-500k BeadChip (Illumina, San Diego, CA, USA), with standard quality control procedures performed on all eligible individuals. Briefly, excluded were samples with <95% completion and SNP assays with call rates <95% or deviating from Hardy–Weinberg equilibrium (*P* < 10^−6^). Only SNPs with minor allele frequencies (MAFs) ≥ 0.05 mapping to autosomal chromosomes were included in the analysis. A total of 416,861 SNPs passed quality control^40^.

Genome-wide imputation following the Michigan Imputation Server pipeline^41^ was performed to estimate missing genotype information. We phased haplotypes with Eagle v2.3 using 1000 Genomes Project data (phase 3) as a reference panel^42^ and then performed imputations using the Minimac (version 3) software. SNPs with an imputation quality score *R*^2^ < 0.8, MAF < 0.05, or *P* < 10^−6^ for the Hardy–Weinberg equilibrium test were excluded from analyses.

### Two-stage GWAS survival analysis

**Fig. 5:**
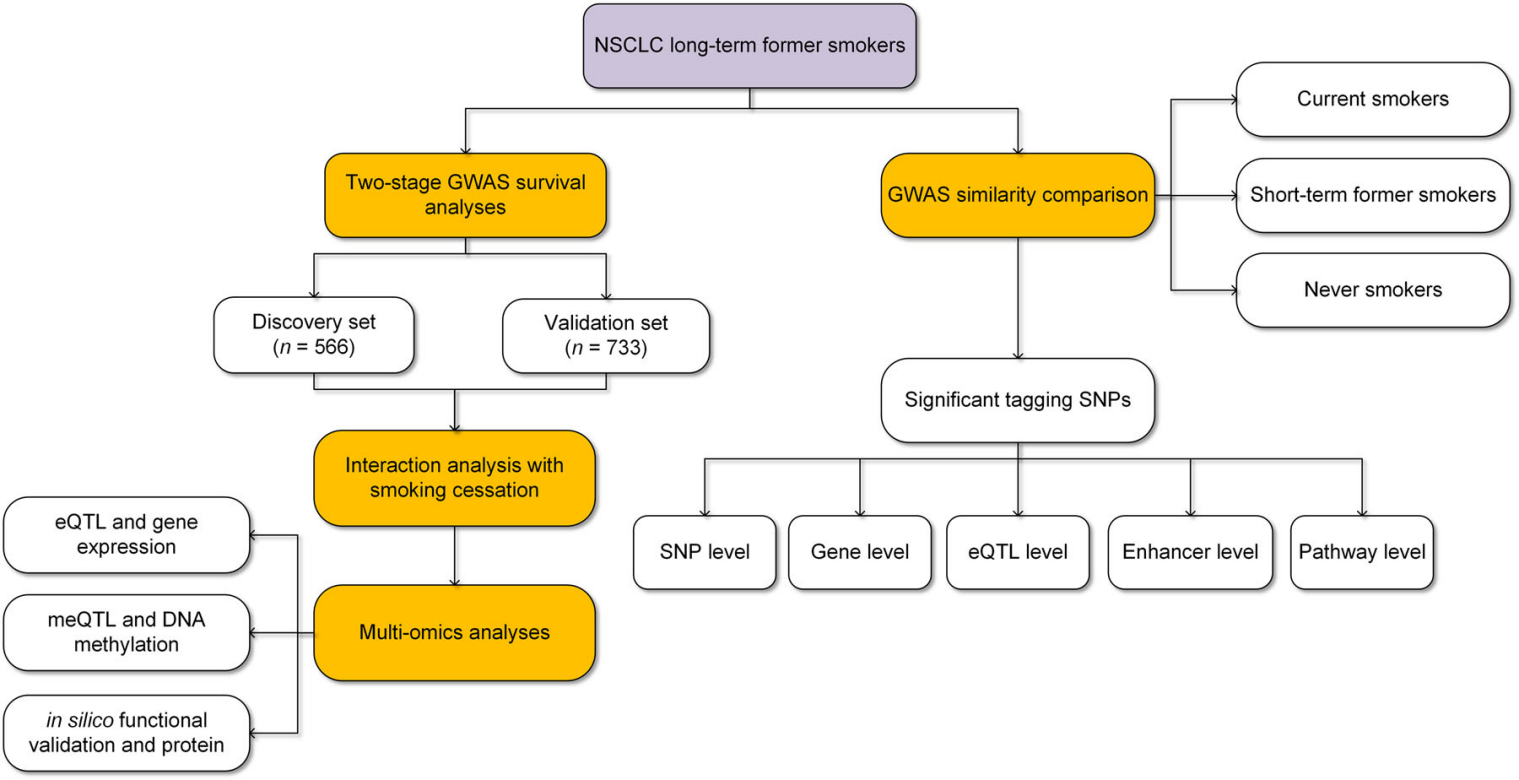
Study workflow. Our study mainly included three parts: (1) GWAS survival study for long-term former smokers; (2) multi-omics study for candidate SNPs and genes; and (3) genetic similarity comparative analysis among different smoking status subgroups.

We used a two-stage strategy to identify significant prognostic SNPs for NSCLC patients who were long-term former smokers (Fig. 5). SNPs were encoded with an additive model (0: wild type; 1: heterozygosity; 2: homozygosity), unless otherwise stated. To quantify the association of each SNP with survival, we used the Cox proportional hazards regression model to evaluate its effect on survival, after adjusting for age, gender, clinical stage (I–IV), histology, pack-years of smoking, years of smoking cessation, and study center. Hazard ratio (HR) and 95% confidence interval (CI) were described for mortality risk for patients per minor allele carried. To control for the confounding effects of population stratification, we also performed principal component analysis (PCA) and included the first three principal components in the model, although no population stratification was observed in the PCA plot (Supplementary Fig. 2). Following the same selection criteria commonly used in the previous GWAS survival studies of cancers^43,44^, we defined significant SNPs as those that met the following criteria: (i) *P* ≤ 10^−5^ in the discovery set; (ii) *P* ≤ 0.05 in the validation set; and (iii) *P* ≤ 5×10^−8^ in the combined set, reaching genome-wide significance. Kaplan-Meier curves were generated to illustrate survival differences between groups with different SNP genotypes, DNA methylation levels or gene expression levels.

### Gene–environment interaction analysis

Since these SNPs significantly associated with prognosis of long-term former smokers with NSCLC, we further conducted gene–environment interaction analysis by estimating how their effects varied with increased years of smoking cessation. Specifically, we included the interaction terms between these SNPs and years of smoking cessation in the Cox proportional hazards regression model adjusted for the same covariates aforementioned.

### Multi-omics analysis

We evaluated gene expression, DNA methylation of these significant SNPs, and further performed in silico function prediction and protein analysis.

To examine the eQTL relationships between SNPs and the expression levels of the corresponding genes, we evaluated their associations by using both the summary-level data from 383 lung tissue samples through the GTEx portal (V7 release)^45^ and the individual-level data from 208 NSCLC Caucasian patients who were long-term former smokers in The Cancer Genome Atlas (TCGA) database (Supplementary Table 8). The cis-eQTL information in GTEx including normalized effect size, standard error, and nominal *p* value for each SNP-expression pair was collected. Genotype data from TCGA were collected from the GDC Legacy Data Portal (Level 2, birdseed data), which were generated from Affymetrix Genome-Wide Human SNP 6.0 Array. We performed the same quality control and imputation procedures as aforementioned. Gene expression values were normalized using the RNA-seq by expectation–maximization method^46^, and dichotomized, when needed, into low- and high-expression subgroups by the median values. To summarize the eQTL effects from TCGA and GTEx, we used meta-analysis with the fixed-effects model. The Cox proportional hazards regression model adjusted for the same covariates as aforementioned was utilized to evaluate the prognostic effects of gene expressions in tumor tissues in TCGA.

The association between SNP and DNA methylation was tested among 155 Caucasian patients who were long-term former smokers in TCGA. DNA methylation data were profiled using Illumina HumanMethylation450 BeadChips. The details of quality control were described in^47^. We used the linear regression model to assess meQTL effects and the Cox proportional hazards regression model to evaluate the association between methylation CpG probes (dichotomized by the median values) and survival. These models were adjusted for the same covariates. False discovery rate adjusted *P* value (*q* value) was used to correct for multiple comparisons.

We used an in silico approach through SNPinfo^48^, RegulomeDB^49^, and HaploReg v4.1^50^ to predict potential functions of the identified SNPs. We also compared protein levels among the 101 tumors paired with normal adjacent lung cancer tissue samples using Student’s paired *t* test in the Clinical Proteomic Tumor Analysis Consortium (CPTAC) project^51^. The data were normalized following the CPTAC Common Data Analysis Pipeline.

### Genetic similarity comparative analysis

To assess genetic similarity of long-term former smoking patients with other smoking subgroups (e.g., never, short-term former, and current smokers), we performed GWAS survival analysis within each smoking subgroup using the Cox proportional hazards regression model adjusted for the same covariates aforementioned. We selected SNPs with moderate association strengths (*P* < 1 × 10^−3^)^36^. Genetic similarity comparisons were made at the SNP, gene, eQTL, enhancer, and pathway levels, respectively.

For gene-level analyses, germline-regulated genes were defined as the nearest genes using the Ensembl definitions on genome build GRCh37 (hg19), which annotated the genomic locations for each subgroup using gene start and stop coordinates^52^. All gene features were defined by GENCODE V25^53^. We kept SNPs with no strong linkage disequilibrium (LD) using the PLINK *indep-pairwise* function; the *r*^2^ used for all LD trimming was 0.5. eQTLs were collected from GTEx lung tissues as described above.

For enhancer-level analysis, the FANTOM5 human enhancer database was used to identify enhancer activities across most cell types and tissues^54^. SNPs located within a permissive enhancer region ±1 kb were defined as the enhancer-related variants.

For pathway-level analysis, we performed gene enrichment pathway analysis based on the KEGG database. All enrichment analyses were performed using the R package *clusterProfiler*^55^.

All statistical analyses were performed using R (v3.5.2) or PLINK (v1.9).

### Reporting summary

Further information on research design is available in the Nature Research Reporting Summary linked to this article.

## Supplementary information

Supplementary Information

Reporting Summary

## Data Availability

The data generated and analyzed during this study are described in the following data record: 10.6084/m9.figshare.14229347^56^. This study utilized subsets of the Oncoarray Consortium—Lung Cancer Studies data available from the dpGap repository: https://identifiers.org/dbgap:phs001273.v3.p2^57^. Specifically data from CAPUA study (CARET), the Roy Castle Lung Study (Liverpool Lung Cancer Project), the M.D. Anderson Cancer Center Study (MDACC study), the Mount-Sinai Hospital-Princess Margaret Study (MSH-PMH), and the Harvard Lung Cancer Study (HLCS) were accessed for this study. Prospective users of these data must apply for access, and details of how to apply can be found on the dataset landing page. Functional prediction analyses of rs34211819 and rs1143149 (supporting Supplementary Table 4) are available from the HaploReg website https://pubs.broadinstitute.org/mammals/haploreg/haploreg.php. De-identified participant demographic and phenotype data for lung adenocarcinoma patients are available from the National Cancer Institute GDC Legacy Archive https://portal.gdc.cancer.gov/legacy-archive/files/0f56f656-18f5-4648-97d9-bdbb0f2184a2. The CPTAC proteomics data file (CPTAC_Pro.xlsx) can be openly accessed from the NCI Cancer Research Data Commons repository https://proteomic.datacommons.cancer.gov/pdc/study/PDC000153. The eQTL data (GTEx_Analysis_v8_eQTL.tar) can be accessed directly from https://storage.googleapis.com/gtex_analysis_v8/single_tissue_qtl_data/GTEx_Analysis_v8_eQTL.tar.

## Code availability

The code that supports the findings of this study is available from the corresponding author on reasonable request.
