# Supplementary material for: A multi-omics study links TNS3 and SEPT7 to long-term former smoking NSCLC survival
Source: NPJ Precis Oncol. 2021 May 17;5:39. doi: 10.1038/s41698-021-00182-3 (PMC8128887; doi:10.1038/s41698-021-00182-3)
Supplement: Supplementary file 1 — Supplementary Information [file 41698_2021_182_MOESM1_ESM.pdf]

Supplementary Information

Study Populations

Discovery set:

CAPUA. The CAncer de PUlmon en Asturias (CAPUA study) is a hospital-based case-control study conducted in Asturias, Spain by the University of Oviedo. Lung cancer cases were recruited from three main hospitals of Asturias in 2002–2012, following an identical protocol. Eligible cases were incident cases of histologically confirmed lung cancer in patients 30-85 years of age who were residents in the geographical area of each participating hospital. Epidemiologic data were collected personally through computer-assisted questionnaires by trained interviewers during the first hospital admission. Structured questionnaires collected information on sociodemographic characteristics, recent and prior tobacco use, environmental exposure (air pollution and passive smoking), diet, personal and family history of cancer, and occupational history from each participant. Peripheral blood samples (or mouthwash samples when they refused to donate blood) were collected from all participants. Coding of histology was based on 2001 WHO/IASLC. Genomic DNA was extracted based on a standard protocol.

CARET. The Carotene and Retinol Efficacy Trial (CARET) was a randomized, double-blind, placebo-controlled trial of the cancer prevention efficacy and safety of a daily combination of 30 mg beta-carotene and 25,000 IU retinyl palmitate in 18,314 persons at high risk for lung cancer. CARET began in 1985, and the intervention was halted in January 1996, 21 months ahead of schedule, with twin conclusions of

definitive evidence of no benefit and substantial evidence of a harmful effect of the intervention on both lung cancer incidence and total mortality. CARET continued to follow and collect endpoints on participants through 2005. Pathology reports and medical records were reviewed to confirm cancer endpoints, and death certificates were obtained to capture the cause of death. During the active intervention phase of CARET, serum, plasma, whole blood, and lung tissue specimens were collected from participants. These biospecimens make up the CARET Biorepository. For the OncoArray Project, CARET provided DNA extracted from the whole blood of lung cancer cases and age-matched controls at baseline (±4 years) and information on sex, race, baseline smoking status, history of occupational asbestos exposure (asbestos vs heavy smoker), and year of enrollment (2-year intervals).

Liverpool. The Liverpool Lung Project is a case-control and cohort study that has recruited >11,500 individuals since 1996 from the Liverpool region in the UK. Detailed epidemiological and clinical data have been collected with associated specimens (i.e., tumor tissue, blood, plasma, sputum, bronchial lavage, and oral brushings). Participants completed a detailed lifestyle questionnaire at recruitment, with repeat questionnaires at intervals; updated data on clinical outcome and hospital events have been collected through the Health and Social Care Information Centre (including Office of National Statistics mortality data, Cancer Registry and Health Episode Statistics). The project was registered in the UK National Institute for Health Research lung cancer portfolio and received the required ethical approvals and sponsorship arrangements. Lung tumors were reviewed by a reference pathologist.

MDACC. Lung cancer cases and frequency-matched controls were ascertained from a large ongoing case-control study at the University of Texas MD Anderson Cancer Center (UTMDACC) that began in 1991. Detailed study description was provided previously (1). In brief, cases were newly diagnosed and histologically confirmed lung cancer patients recruited from UTMDACC. After providing written informed consent, each study participant completed an in-person interview with staff to collect information on demographics, smoking status, etc. Blood samples were also drawn from all study participants. This study was approved by institutional review boards of UTMDACC and Kelsey-Seybold Clinics.

The Mount‐Sinai Hospital‐Princess Margaret Study (MSH-PMH). MSH-PMH was conducted in the greater Toronto area from 2008-2013. Lung cancer cases were recruited from hospitals in the network of the University of Toronto. All subjects were interviewed, and information on lifestyle risk factors, occupational history, and medical and family history was collected using a standard questionnaire. Tumors were centrally reviewed by a reference pathologist [a member of the International Association for the Study of Lung Cancer (IASLC) committee] and a second pathologist in the University Health Network. If the reviews conflicted, a consensus was reached after discussion. Coding of histology was based on 2001 WHO/IASLC. Genomic DNA was extracted based on a standard protocol.

Validation set:

The Harvard Lung Cancer Study (HLCS). HLCS is a case-control study based at Mass General Hospital (MGH) in Boston, Massachusetts from 1992-2004. Details of

the study were described previously (2). Briefly, eligible cases included any person >18 years old with a diagnosis of primary lung cancer that was further confirmed by an MGH lung pathologist. Interviewer-administered questionnaires and a modified version of the standardized American Thoracic Society respiratory questionnaire were completed, and information on demographics, medical history, family history of cancer, smoking history, and detailed work history, including job titles and tasks, was collected. The Institutional Review Board of MGH and the Human Subjects Committee of the Harvard School of Public Health approved the study.

References

1. Spitz MR, Hong WK, Amos CI, Wu X, Schabath MB, Dong Q, et al. A risk model for prediction of lung cancer. Journal of the National Cancer Institute. 2007;99(9):715-26.
2. Huang Y-T, Heist RS, Chirieac LR, Lin X, Skaug V, Zienolddiny S, et al. Genome-wide analysis of survival in early-stage non–small-cell lung cancer. Journal of clinical oncology. 2009;27(16):2660.

Supplementary Figure 1. Quantile-quantile plot of results in the genome-wide survival analysis of NSCLC patients who were long-term former smokers in the combined set.

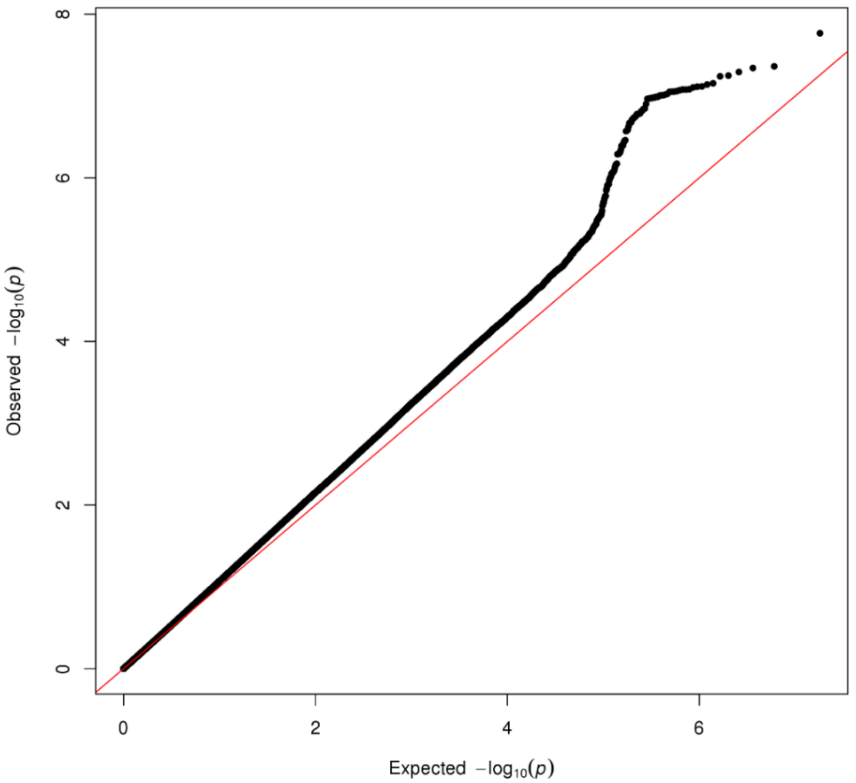

Supplementary Figure 2. Population stratification plot for NSCLC patients who have genome-wide SNP genotypes in our study.

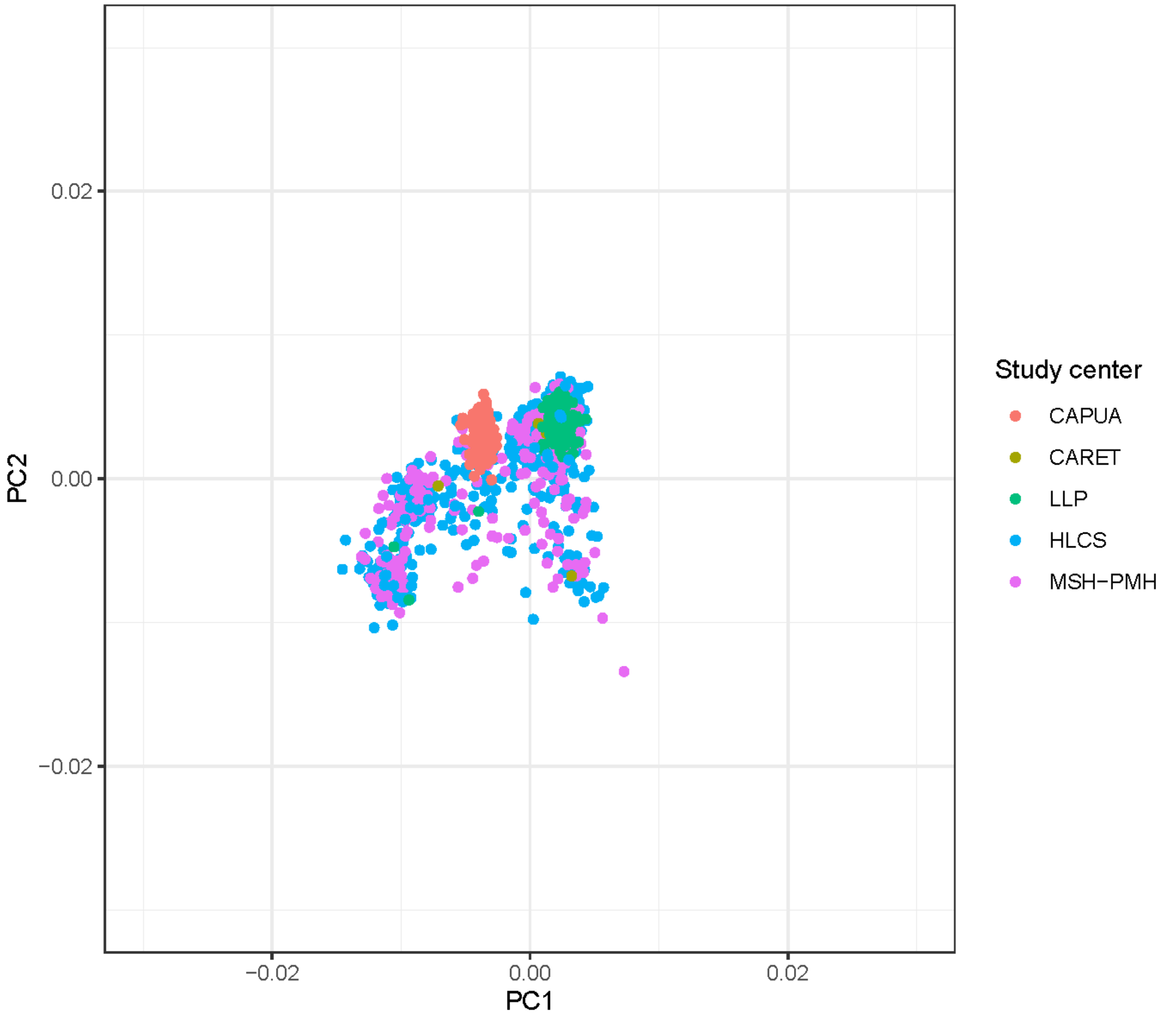

Supplementary Table 1. Association results for SNPs within *TNS3* and *SEPT7* regions derived from Cox proportional hazards regression model adjusted for covariates in NSCLC patients who were long-term former smokers.

SNP	LD-r^2	MAF	Position	HR	SE	*P*	Gene	Consequence
**rs34211819**	**1.00**	**0.35**	**47539511**	**0.74**	**0.05**	**3.9E-09**	***TNS3***	**intron_variant**
rs334496	0.64	0.28	47546375	0.72	0.06	6.7E-09	*TNS3*	intron_variant
rs11977711	0.66	0.27	47560950	0.73	0.06	2.15E-08	*TNS3*	intron_variant
rs334528	0.60	0.30	47555649	0.75	0.05	1.33E-07	*TNS3*	intron_variant
rs11973266	0.68	0.28	47551373	0.75	0.06	2.07E-07	*TNS3*	intron_variant
rs62448395	0.66	0.27	47563713	0.75	0.06	2.1E-07	*TNS3*	intron_variant
rs614280	0.63	0.29	47553902	0.76	0.05	3.71E-07	*TNS3*	intron_variant
rs6942844	0.83	0.33	47539700	0.77	0.05	4.64E-07	*TNS3*	intron_variant
rs334502	0.64	0.29	47549108	0.76	0.05	5.8E-07	*TNS3*	intron_variant
rs11973279	0.68	0.28	47551206	0.76	0.06	8.04E-07	*TNS3*	intron_variant
rs615147	0.63	0.29	47554079	0.77	0.05	0.000001	*TNS3*	intron_variant
rs6948207	0.98	0.35	47540878	0.78	0.05	1.21E-06	*TNS3*	intron_variant
rs34387805	0.67	0.29	47544862	0.77	0.05	2.08E-06	*TNS3*	intron_variant
rs11974349	0.70	0.29	47543452	0.77	0.05	2.76E-06	*TNS3*	intron_variant
rs11342271	0.70	0.28	47544590	0.78	0.06	4.36E-06	*TNS3*	intron_variant&feature_truncation
rs1009701	0.70	0.29	47543793	0.77	0.06	5.15E-06	*TNS3*	intron_variant
rs6947181	0.99	0.35	47540088	0.80	0.05	1.41E-05	*TNS3*	intron_variant
rs334499	0.64	0.29	47547215	0.80	0.05	2.24E-05	*TNS3*	intron_variant

rs884972	0.81	0.31	47537679	0.80	0.05	3.22E-05	*TNS3*	intron_variant
rs2271312	0.82	0.31	47538547	0.80	0.05	0.000037	*TNS3*	intron_variant
rs35267224	0.69	0.29	47543041	0.80	0.06	4.49E-05	*TNS3*	intron_variant
**rs1143149**	**1.00**	**0.34**	**35928275**	**1.36**	**0.05**	**9.75E-09**	***SEPT7***	**intron_variant**
rs10227145	0.70	0.32	35838147	1.31	0.05	2.54E-07	*SEPT7*	upstream_gene_variant
rs12666886	0.84	0.33	35846108	1.31	0.05	2.66E-07	*SEPT7*	intron_variant
rs10260003	0.84	0.33	35868867	1.31	0.05	2.71E-07	*SEPT7*	intron_variant
rs9648424	0.84	0.33	35873540	1.31	0.05	3.09E-07	*SEPT7*	intron_variant
rs12701464	0.89	0.33	35888034	1.30	0.05	3.34E-07	*SEPT7*	intron_variant
rs10214937	0.82	0.33	35862144	1.30	0.05	3.35E-07	*SEPT7*	intron_variant
rs1420642	0.84	0.33	35870386	1.30	0.05	3.85E-07	*SEPT7*	intron_variant
rs6979336	0.84	0.33	35847041	1.30	0.05	3.93E-07	*SEPT7*	intron_variant
rs10255768	0.93	0.33	35897955	1.30	0.05	4.08E-07	*SEPT7*	intron_variant
rs35244784	0.93	0.33	35878438	1.30	0.05	4.34E-07	*SEPT7*	intron_variant
rs6462629	0.91	0.33	35884997	1.30	0.05	4.34E-07	*SEPT7*	intron_variant
rs6947319	0.71	0.33	35841297	1.30	0.05	4.5E-07	*SEPT7*	intron_variant
rs9690709	0.93	0.33	35896296	1.30	0.05	4.72E-07	*SEPT7*	intron_variant
rs6964244	0.84	0.33	35861799	1.30	0.05	4.93E-07	*SEPT7*	intron_variant
rs10254993	0.84	0.33	35850222	1.30	0.05	5.19E-07	*SEPT7*	intron_variant
rs35769007	0.87	0.33	35889141	1.30	0.05	5.19E-07	*SEPT7*	intron_variant
rs6462630	0.93	0.33	35886257	1.30	0.05	5.26E-07	*SEPT7*	intron_variant

rs2893514	0.84	0.33	35856339	1.30	0.05	5.36E-07	*SEPT7*	intron_variant
rs13233646	0.93	0.33	35882979	1.30	0.05	5.36E-07	*SEPT7*	intron_variant
rs13220938	0.93	0.33	35883044	1.30	0.05	5.36E-07	*SEPT7*	intron_variant
rs12701463	0.91	0.33	35885574	1.30	0.05	5.36E-07	*SEPT7*	intron_variant
rs12701459	0.81	0.33	35847216	1.30	0.05	6.21E-07	*SEPT7*	intron_variant
rs10232178	0.93	0.33	35894239	1.30	0.05	6.54E-07	*SEPT7*	intron_variant
rs12701462	0.93	0.33	35884296	1.30	0.05	6.57E-07	*SEPT7*	intron_variant
rs6953271	0.84	0.33	35859661	1.30	0.05	7.22E-07	*SEPT7*	intron_variant
rs2715605	0.65	0.40	35904932	1.28	0.05	7.86E-07	*SEPT7*	intron_variant
rs2727853	0.65	0.41	35880696	1.28	0.05	8.93E-07	*SEPT7*	intron_variant
rs2715604	0.65	0.40	35904380	1.28	0.05	9.44E-07	*SEPT7*	intron_variant
rs7805632	0.95	0.34	35947484	1.30	0.05	9.46E-07	*SEPT7*	downstream_gene_variant
rs13234117	0.91	0.33	35883401	1.29	0.05	9.48E-07	*SEPT7*	intron_variant
rs13225904	0.93	0.34	35908733	1.29	0.05	9.58E-07	*SEPT7*	intron_variant
rs7809390	0.96	0.34	35939715	1.30	0.05	1.02E-06	*SEPT7*	intron_variant
rs56287072	0.65	0.40	35898790	1.28	0.05	1.02E-06	*SEPT7*	intron_variant
rs13437988	0.94	0.33	35901490	1.29	0.05	1.08E-06	*SEPT7*	intron_variant
rs7789421	0.97	0.34	35943447	1.30	0.05	1.09E-06	*SEPT7*	intron_variant
rs13437979	0.94	0.33	35901524	1.29	0.05	1.15E-06	*SEPT7*	intron_variant
rs6955933	0.94	0.34	35913034	1.29	0.05	1.36E-06	*SEPT7*	intron_variant
rs2249906	0.65	0.41	35879599	1.27	0.05	1.6E-06	*SEPT7*	intron_variant

rs13244180	1.00	0.34	35925878	1.30	0.05	1.63E-06	*SEPT7*	intron_variant
rs9638914	0.99	0.34	35920755	1.29	0.05	1.73E-06	*SEPT7*	intron_variant
rs2541306	0.63	0.42	35878931	1.27	0.05	2.35E-06	*SEPT7*	intron_variant
rs10235020	0.93	0.33	35900104	1.28	0.05	2.36E-06	*SEPT7*	intron_variant
rs6945909	1.00	0.34	35928551	1.29	0.05	2.65E-06	*SEPT7*	intron_variant
rs10244144	0.99	0.34	35930437	1.28	0.05	4.17E-06	*SEPT7*	intron_variant
rs28522781	1.00	0.34	35930260	1.28	0.05	4.32E-06	*SEPT7*	intron_variant
rs2715598	0.65	0.42	35891893	1.26	0.05	4.63E-06	*SEPT7*	intron_variant
rs10233082	0.95	0.34	35946779	1.29	0.06	4.72E-06	*SEPT7*	downstream_gene_variant
rs4445132	0.94	0.34	35913113	1.28	0.05	4.79E-06	*SEPT7*	intron_variant
rs4723450	0.68	0.40	35948676	1.26	0.05	5.11E-06	*SEPT7*	downstream_gene_variant
rs2715595	0.65	0.41	35908875	1.26	0.05	5.26E-06	*SEPT7*	intron_variant
rs2727845	0.69	0.40	35921378	1.26	0.05	5.68E-06	*SEPT7*	intron_variant
rs2727850	0.65	0.42	35894737	1.26	0.05	6.69E-06	*SEPT7*	intron_variant
rs56231318	0.69	0.40	35935498	1.25	0.05	6.86E-06	*SEPT7*	intron_variant
rs7792970	0.65	0.40	35906517	1.26	0.05	7.87E-06	*SEPT7*	intron_variant
rs62452859	0.69	0.40	35920270	1.26	0.05	8.87E-06	*SEPT7*	intron_variant
rs13232794	0.91	0.35	35914049	1.27	0.05	0.00001	*SEPT7*	intron_variant
rs10276852	1.00	0.34	35930569	1.26	0.05	1.06E-05	*SEPT7*	intron_variant
rs2727859	0.69	0.40	35918407	1.25	0.05	1.09E-05	*SEPT7*	intron_variant
rs4131719	0.99	0.34	35938580	1.27	0.05	1.26E-05	*SEPT7*	intron_variant

rs3779233	0.97	0.34	35921770	1.27	0.05	1.26E-05	*SEPT7*	intron_variant
rs3801339	0.68	0.39	35947810	1.25	0.05	0.000015	*SEPT7*	downstream_gene_variant
rs3890242	0.66	0.41	35909701	1.24	0.05	1.71E-05	*SEPT7*	intron_variant
rs2710801	0.68	0.40	35940306	1.25	0.05	1.74E-05	*SEPT7*	intron_variant
rs2727854	0.70	0.40	35926142	1.24	0.05	0.000019	*SEPT7*	intron_variant
rs10230802	0.99	0.34	35933953	1.25	0.05	2.49E-05	*SEPT7*	intron_variant
rs6462633	0.99	0.34	35919741	1.25	0.05	2.72E-05	*SEPT7*	intron_variant
rs6955154	0.65	0.40	35912637	1.23	0.05	3.17E-05	*SEPT7*	intron_variant
rs2727856	0.70	0.40	35932266	1.24	0.05	4.41E-05	*SEPT7*	intron_variant
rs58918750	0.70	0.40	35928678	1.23	0.05	4.63E-05	*SEPT7*	intron_variant
rs3801336	0.94	0.34	35913562	1.24	0.05	5.15E-05	*SEPT7*	intron_variant
rs3801337	0.94	0.34	35913564	1.24	0.05	5.77E-05	*SEPT7*	intron_variant
rs13221258	1.00	0.34	35927385	1.24	0.05	5.89E-05	*SEPT7*	intron_variant
rs62454465	0.68	0.40	35939361	1.23	0.05	6.21E-05	*SEPT7*	intron_variant
rs4720187	0.69	0.40	35938891	1.23	0.05	6.73E-05	*SEPT7*	intron_variant
rs4723449	0.68	0.40	35916909	1.22	0.05	6.77E-05	*SEPT7*	intron_variant
rs55904294	0.69	0.40	35933862	1.22	0.05	6.85E-05	*SEPT7*	intron_variant
rs67144743	0.69	0.40	35924156	1.22	0.05	0.0001	*SEPT7*	intron_variant
rs6959502	0.68	0.40	35949021	1.21	0.05	0.000159	*SEPT7*	downstream_gene_variant
rs2392387	0.69	0.40	35923893	1.20	0.05	0.000477	*SEPT7*	intron_variant

SNPs of high LD (r^2>0.6) with *TNS3* or *SEPT7* that had moderate prognostic effect (P < 0.001) are reported in the table.

SNP: single nucleotide polymorphism; NSCLC: non-small cell lung cancer; LD: linkage disequilibrium; MAF: minor allele frequency; HR: hazard ratio; SE: standard error; HR, SE and P values were derived from Cox proportional hazards regression model adjusted for age, gender, clinical stage (I–IV), histology, pack-years of smoking, years of smoking cessation, and study center.

Supplementary Table 2. Association between two identified SNPs and overall survival of NSCLC patients who were long-term former smokers in the combined set using different genetic models.

Genetic model	rs34211819				Genetic model	rs1143149			
	N (deaths)	MST	HR (95% CI)	*P*		*N* (deaths)	MST	HR (95% CI)	*P*
Additive			0.73 (0.66-0.81)	3.90×10^{-9}	Additive			1.36 (1.22-1.51)	9.75×10^{-9}
Co-dominant					Co-dominant				
AA	511 (370)	2.41	Reference		GG	544 (362)	2.80	Reference	
AC	581 (393)	2.63	0.82 (0.71-0.95)	0.009	CG	561 (385)	2.69	1.34 (1.15-1.56)	1.27×10^{-4}
CC	165 (101)	3.17	0.49 (0.38-0.62)	2.13×10^{-9}	CC	132 (98)	1.49	1.89 (1.50-2.38)	5.96×10^{-8}
Dominant					Dominant				
AA	511 (370)	2.41	Reference		GG	544 (362)	2.80	Reference	
AC/CC	746 (494)	2.76	0.73 (0.63-0.84)	1.13×10^{-5}	CG/CC	693 (483)	2.33	1.42 (1.23-1.64)	1.20×10^{-6}
Recessive					Recessive				
AC/AA	1092 (763)	2.52	Reference		CG/GG	1105 (747)	2.75	Reference	
CC	165 (101)	3.17	0.54 (0.43-0.67)	6.45×10^{-8}	CC	132 (98)	1.49	1.63 (1.31-2.02)	9.16×10^{-6}

SNP: single nucleotide polymorphism; NSCLC: non-small cell lung cancer; MST: median survival time (years); HR: hazard ratio; CI: confidence interval; HR, 95% CI and P values were derived from the Cox proportional hazards regression model adjusted for age, gender, clinical stage (I–IV), histology, pack-years of smoking, years of smoking cessation, and study center.

Supplementary Table 3. Association results derived from DNA meQTL analysis and survival analysis for *TNS3* and *SEPT7*.

Gene	Name	CHR	Position	Gene Group	CpG Island	meQTL (95%CI)	*FDR-q*	HR (95%CI)	*FDR-q*
TNS3	cg22455271	7	47333085	Body	NA	-0.08 (-0.14~-0.01)	0.034	2.40 (1.47-3.93)	0.001
SEPT7	cg07462932	7	35840534	TSS200	Island	-0.17 (-0.32~-0.02)	0.039	0.47 (0.30-0.74)	0.018

CHR: chromosome; meQTL: methylation quantitative trait loci; FDR: false discovery rate; HR: hazard ratio; CI: confidence interval; NA: not available.

Supplementary Table 4. Functional prediction analyses of rs34211819 and rs1143149.

SNP	Gene	CHR	Allele	Regulome DB Rank	Promoter histone marks	Enhancer histone marks	DNase	Proteins bound	Motifs changed	Function annotation
rs34211819	TNS3	7	C/A	3a	LIV, GI	15 tissues	LNG, BRN	BCL3, OCT2	Maf	intronic
rs1143149	SEPT7	7	C/G	5					Mef2, SIX5	intronic

SNP: single nucleotide polymorphism; CHR: chromosome

Supplementary Table 5. Genes shared by different smoking subgroups.

CHR	Gene type	Gene symbol	Gene ID	CHR	Gene type	Gene symbol	Gene ID
1	protein_coding	*PRDM16*	ENSG00000142611	7	protein_coding	CREB5	ENSG00000146592
1	protein_coding	*CAMTA1*	ENSG00000171735	7	protein_coding	AMPH	ENSG00000078053
1	protein_coding	*KAZN*	ENSG00000189337	7	protein_coding	ABCA13	ENSG00000179869
1	protein_coding	*IGSF21*	ENSG00000117154	7	protein_coding	AUTS2	ENSG00000158321
1	protein_coding	*PAX7*	ENSG00000009709	7	protein_coding	CALN1	ENSG00000183166
1	protein_coding	*CSMD2*	ENSG00000121904	7	protein_coding	MAGI2	ENSG00000187391
1	protein_coding	*PPIE*	ENSG00000084072	7	protein_coding	CDK14	ENSG00000058091
1	protein_coding	*HIVEP3*	ENSG00000127124	7	protein_coding	CDHR3	ENSG00000128536
1	protein_coding	*LRRC7*	ENSG00000033122	7	protein_coding	CNTNAP2	ENSG00000174469
1	protein_coding	*ST6GALNAC3*	ENSG00000184005	7	protein_coding	DPP6	ENSG00000130226
1	protein_coding	*DPYD*	ENSG00000188641	7	protein_coding	PTPRN2	ENSG00000155093
1	protein_coding	*LMX1A*	ENSG00000162761	8	protein_coding	CSMD1	ENSG00000183117
1	protein_coding	*TARBP1*	ENSG00000059588	8	protein_coding	SGCZ	ENSG00000185053
1	protein_coding	*RYR2*	ENSG00000198626	8	protein_coding	PSD3	ENSG00000156011
1	protein_coding	*KIF26B*	ENSG00000162849	8	antisense	RP11-624C23.1	ENSG00000253535
2	lincRNA	*MIR3681HG*	ENSG00000224184	8	protein_coding	UNC5D	ENSG00000156687
2	protein_coding	*ALK*	ENSG00000171094	8	protein_coding	ZMAT4	ENSG00000165061
2	antisense	*SLC8A1-AS1*	ENSG00000227028	8	protein_coding	TOX	ENSG00000198846
2	protein_coding	*PRKCE*	ENSG00000171132	8	protein_coding	KCNB2	ENSG00000182674
2	lincRNA	*AC007682.1*	ENSG00000231918	8	protein_coding	ADGRB1	ENSG00000181790
2	protein_coding	*ACYP2*	ENSG00000170634	9	protein_coding	GLIS3	ENSG00000107249
2	protein_coding	*EML6*	ENSG00000214595	9	protein_coding	PTPRD	ENSG00000153707

2	lincRNA	*RP11-444A22.1*	ENSG00000271955	9	protein_coding	TEK	ENSG00000120156
2	processed_transcript	*AC074391.1*	ENSG00000204929	9	protein_coding	LINGO2	ENSG00000174482
2	protein_coding	*LRRTM4*	ENSG00000176204	9	protein_coding	PCSK5	ENSG00000099139
2	protein_coding	*CTNNA2*	ENSG00000066032	9	protein_coding	PALM2-AKAP2	ENSG00000157654
2	protein_coding	*DPP10*	ENSG00000175497	10	protein_coding	CELF2	ENSG00000048740
2	protein_coding	*THSD7B*	ENSG00000144229	10	protein_coding	CAMK1D	ENSG00000183049
2	protein_coding	*LRP1B*	ENSG00000168702	10	protein_coding	CACNB2	ENSG00000165995
2	protein_coding	*ARHGAP15*	ENSG00000075884	10	protein_coding	KIAA1217	ENSG00000120549
2	protein_coding	*GALNT13*	ENSG00000144278	10	protein_coding	PCDH15	ENSG00000150275
2	protein_coding	*SPAG16*	ENSG00000144451	10	protein_coding	CTNNA3	ENSG00000183230
3	protein_coding	*CNTN4*	ENSG00000144619	10	protein_coding	HPSE2	ENSG00000172987
3	protein_coding	*SUMF1*	ENSG00000144455	10	protein_coding	SORCS3	ENSG00000156395
3	protein_coding	*ITPR1*	ENSG00000150995	10	lincRNA	LINC01435	ENSG00000229981
3	protein_coding	*GRM7*	ENSG00000196277	10	protein_coding	TACC2	ENSG00000138162
3	antisense	*LMCD1-AS1*	ENSG00000227110	10	protein_coding	ADAM12	ENSG00000148848
3	protein_coding	*IQSEC1*	ENSG00000144711	11	protein_coding	NAV2	ENSG00000166833
3	protein_coding	*TBC1D5*	ENSG00000131374	11	protein_coding	NELL1	ENSG00000165973
3	protein_coding	*RBMS3*	ENSG00000144642	11	protein_coding	ABTB2	ENSG00000166016
3	protein_coding	*CACNA1D*	ENSG00000157388	11	protein_coding	LRRC4C	ENSG00000148948
3	protein_coding	*CACNA2D3*	ENSG00000157445	11	protein_coding	SHANK2	ENSG00000162105
3	protein_coding	*ERC2*	ENSG00000187672	11	protein_coding	DLG2	ENSG00000150672
3	protein_coding	*FHIT*	ENSG00000189283	11	protein_coding	CNTN5	ENSG00000149972
3	protein_coding	*PTPRG*	ENSG00000144724	11	protein_coding	GRIK4	ENSG00000149403

3	protein_coding	*PRICKLE2*	ENSG00000163637	11	protein_coding	NTM	ENSG00000182667
3	protein_coding	*FAM19A1*	ENSG00000183662	11	protein_coding	OPCML	ENSG00000183715
3	protein_coding	*FRMD4B*	ENSG00000114541	12	protein_coding	TSPAN9	ENSG00000011105
3	protein_coding	*PDZRN3*	ENSG00000121440	12	protein_coding	GALNT6	ENSG00000139629
3	protein_coding	*ROBO2*	ENSG00000185008	12	protein_coding	PLXNC1	ENSG00000136040
3	protein_coding	*CADM2*	ENSG00000175161	12	protein_coding	TMEM132B	ENSG00000139364
3	protein_coding	*LSAMP*	ENSG00000185565	12	protein_coding	TMEM132D	ENSG00000151952
3	protein_coding	*CPNE4*	ENSG00000196353	13	protein_coding	SPATA13	ENSG00000182957
3	processed_transcript	*RP11-23D24.2*	ENSG00000238755	13	protein_coding	FARP1	ENSG00000152767
3	protein_coding	*LPP*	ENSG00000145012	13	protein_coding	FAM155A	ENSG00000204442
4	protein_coding	*STK32B*	ENSG00000152953	13	protein_coding	MYO16	ENSG00000041515
4	protein_coding	*SORCS2*	ENSG00000184985	14	transcribed_processed_pseudogene	CTD-3006G17.2	ENSG00000258932
4	protein_coding	*ABLIM2*	ENSG00000163995	14	protein_coding	NPAS3	ENSG00000151322
4	protein_coding	*LDB2*	ENSG00000169744	14	protein_coding	EGLN3	ENSG00000129521
4	protein_coding	*KCNIP4*	ENSG00000185774	15	protein_coding	SNRPN	ENSG00000128739
4	protein_coding	*NWD2*	ENSG00000174145	15	protein_coding	GABRB3	ENSG00000166206
4	protein_coding	*RBM47*	ENSG00000163694	15	protein_coding	RYR3	ENSG00000198838
4	protein_coding	*EPHA5*	ENSG00000145242	15	lincRNA	RP11-624L4.1	ENSG00000259345
4	protein_coding	*GRID2*	ENSG00000152208	15	protein_coding	RORA	ENSG00000069667
4	protein_coding	*UNC5C*	ENSG00000182168	15	protein_coding	THSD4	ENSG00000187720
4	protein_coding	*RNF150*	ENSG00000170153	15	protein_coding	ADAMTSL3	ENSG00000156218
4	protein_coding	*INPP4B*	ENSG00000109452	15	protein_coding	AGBL1	ENSG00000273540
4	protein_coding	*MAR1*	ENSG00000145416	15	protein_coding	SLCO3A1	ENSG00000176463

5	lincRNA	*RP11-122F24.1*	ENSG00000250974	15	lincRNA	CTD-2544M6.1	ENSG00000259199
5	protein_coding	*SEMA5A*	ENSG00000112902	15	protein_coding	IGF1R	ENSG00000140443
5	protein_coding	*CTNND2*	ENSG00000169862	16	protein_coding	RBFOX1	ENSG00000078328
5	protein_coding	*TRIO*	ENSG00000038382	16	protein_coding	SMG1	ENSG00000157106
5	protein_coding	*CDH18*	ENSG00000145526	16	protein_coding	HS3ST4	ENSG00000182601
5	protein_coding	*PDZD2*	ENSG00000133401	16	protein_coding	FTO	ENSG00000140718
5	protein_coding	*RICTOR*	ENSG00000164327	16	protein_coding	WWOX	ENSG00000186153
5	protein_coding	*RAB3C*	ENSG00000152932	17	protein_coding	ABR	ENSG00000159842
5	protein_coding	*SV2C*	ENSG00000122012	17	protein_coding	TRPV3	ENSG00000167723
5	lincRNA	*RP11-6N13.1*	ENSG00000251574	17	protein_coding	PRKCA	ENSG00000154229
5	lincRNA	*CTC-254B4.1*	ENSG00000251027	17	protein_coding	PITPNC1	ENSG00000154217
5	protein_coding	*GRAMD3*	ENSG00000155324	17	lincRNA	AC061992.2	ENSG00000267737
6	protein_coding	*GMDS*	ENSG00000112699	18	protein_coding	MYOM1	ENSG00000101605
6	protein_coding	*SLC22A23*	ENSG00000137266	18	protein_coding	DLGAP1	ENSG00000170579
6	protein_coding	*OFCC1*	ENSG00000181355	18	protein_coding	LDLRAD4	ENSG00000168675
6	protein_coding	*ATXN1*	ENSG00000124788	19	transcribed_unprocessed_pseudogene	C3P1	ENSG00000167798
6	protein_coding	*CDKAL1*	ENSG00000145996	19	transcribed_unprocessed_pseudogene	CEACAM22P	ENSG00000230666
6	protein_coding	*EYS*	ENSG00000188107	20	protein_coding	MACROD2	ENSG00000172264
6	protein_coding	*SLC35F1*	ENSG00000196376	20	protein_coding	PTPRT	ENSG00000196090
6	protein_coding	*SASH1*	ENSG00000111961	20	protein_coding	CDH4	ENSG00000179242
6	protein_coding	*PARK2*	ENSG00000185345	21	protein_coding	NCAM2	ENSG00000154654
6	protein_coding	*PACRG*	ENSG00000112530	21	protein_coding	RUNX1	ENSG00000159216

6	protein_coding	*PDE10A*	ENSG00000112541	21	protein_coding	DSCAM	ENSG00000171587
6	protein_coding	*RPS6KA2*	ENSG00000071242	1	misc_RNA	Y_RNA	ENSG00000200344

CHR: chromosome

Supplementary Table 6. Sensitivity analysis of genetic similarity comparative analysis under different threshold.

Threshold	Smoking subgroup[a]	SNPs	Genes	Protein-coding genes	eQTL genes	Enhancers	KEGG pathways
$P < 10^{-4}$	Never	0	91	74	3	4	4
	Short-term	0	70	58	3	2	4
	Current	0	39	36	2	1	2
	All	0	3	3	0	0	0
$P < 10^{-5}$	Never	0	10	8	0	1	1
	Short-term	0	2	2	0	0	1
	Current	0	1	1	0	0	0
	All	0	0	0	0	0	0

[a]The frequency in each smoking subgroup is the number of shared biomarkers compared with long-term former smoking subgroup.

Supplementary Table 7. Demographic and clinical characteristics of NSCLC patients among different subgroups of smoking status.

Characteristics	Never smokers	Long-term former smokers	Short-term former smokers	Current smokers
Sample size	504	1299	687	1861
Death (%)	295 (58.5)	887 (68.2)	510 (74.3)	1288 (69.3)
Median survival years (95% CI)	2.86 (2.41-3.56)	2.64 (2.38-2.86)	2.27 (2.00-2.77)	1.89 (1.71-2.07)
Age (years)	63.4 ± 11.4	70.4 ± 9.0	66.0 ± 9.7	63.1 ± 9.7
Histology (%)				
LUSC	60 (11.9)	331 (25.5)	267 (38.9)	661 (35.5)
LUAD	420 (83.3)	877 (67.5)	374 (54.4)	1027 (55.2)
NSCLC, not specified	24 (4.8)	91 (7.0)	46 (6.7)	173 (9.3)
Gender, male (%)	179 (35.5)	758 (58.4)	422 (61.4)	1042 (56)
Clinical stage (%)				
I	132 (26.2)	465 (35.8)	240 (34.9)	559 (30)
II	30 (6.0)	140 (10.8)	74 (10.8)	211 (11.3)
III	119 (23.6)	296 (22.8)	189 (27.5)	511 (27.5)
IV	206 (40.9)	366 (28.2)	166 (24.2)	533 (28.6)

LUAD: lung adenocarcinoma; LUSC: lung squamous cell carcinoma; NSCLC: non-small cell lung cancer

Supplementary Table 8. Demographic and clinical characteristics of long-term former smoking NSCLC patients in TCGA.

Characteristics	TCGA
Sample size	208
Age (years)	68.69 ± 9.89
Gender, male (%)	133 (63.9)
Histology (%)	
LUSC	82 (39.5)
LUAD	126 (60.5)
NSCLC, not specified	0
Clinical stage (%)	
I	121 (58.1)
II	48 (23.0)
III	29 (13.9)
IV	10 (4.8)
Pack-years of smoking	34.73 ± 23.34
Years of smoking cessation	29.36 ± 9.06
